# Supplementary material for: Simultaneous multifunctional transcriptome engineering by CRISPR RNA scaffold
Source: Nucleic Acids Res. 2023 Jul 3;51(14):e77. doi: 10.1093/nar/gkad547 (PMC10415119; doi:10.1093/nar/gkad547)
Supplement: gkad547_Supplemental_File [file gkad547_supplemental_file.pdf]

Simultaneous multifunctional transcriptome engineering by CRISPR RNA scaffold

Zukai Liu[1,2], Nathaniel Jillette[1], Paul Robson[1,2,3,4], Albert Cheng[1,2,3,4,5*]

[1] The Jackson Laboratory for Genomic Medicine, Farmington, CT 06032, USA

[2] Department of Genetics and Genome Sciences, University of Connecticut Health Center, Farmington, CT 06030, USA

[3] The Jackson Laboratory Cancer Center, Bar Harbor, ME 04609, USA

[4] Institute for Systems Genomics, University of Connecticut Health Center, Farmington, CT 06030, USA

[5] School of Biological and Health Systems Engineering, Arizona State University, Tempe, AZ 85281, USA

* To whom correspondence should be addressed. Tel: +1 602-543-6735 ; Fax: +1 480-727-7624; Email: albert@cheng.bio

SUPPLEMENTARY INFORMATION

Supplementary Figures S1-S17

Supplementary Protein Sequences

Supplementary Table S1: Oligo sequences

Supplementary Table S2: Plasmids on addgene

Supplementary Figures

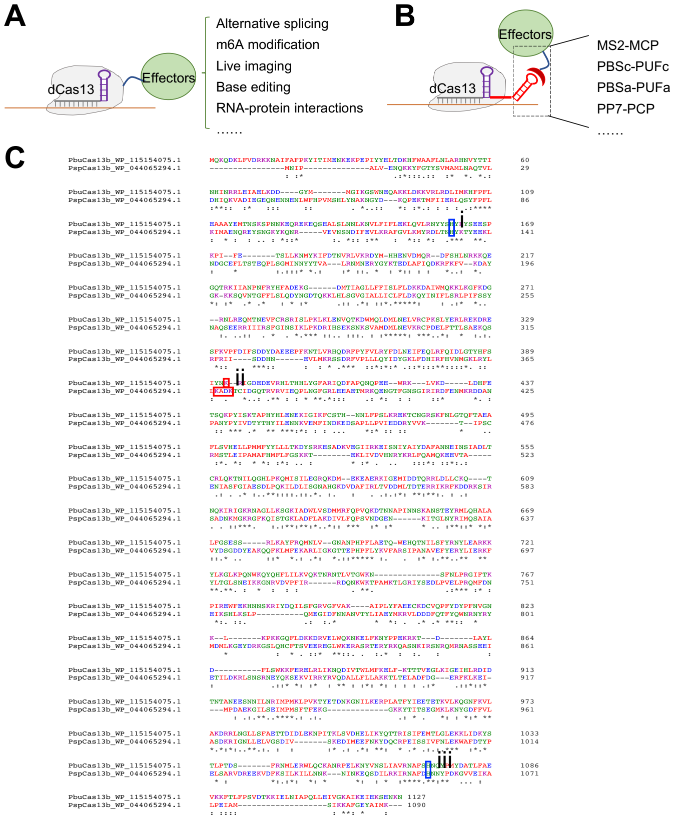

Supplemental figures S1. Combinatorial RNA Editing via Scaffold Tagged gRNA (A) Conventional CRISPR/Cas13 mediated RNA editing. gRNA consists of two parts, the spacer (gray) to match with targeting transcripts and the direct repeats (DR, purple) to bind with Cas13 protein. Different effector can be fused with dCas13 to execute different functions as indicated. (B) In CREST system, one or multiple copies of scaffold RNA motif (red) are added to the 3' end of gRNA and can be recognized and bound by specific RNA binding domains (RBDs). Effectors are fused with RBDs for RNA manipulation. Some scaffold RNA motifs and their cognate RBDs are listed at the right. (C) Alignment of PbuCas13b and PspCas13b. The amino acids in blue rectangle are mutated to alanine to generate the dCas13 and the amino acids in red rectangle are mutated to alanine to disable the crRNA processing activity of Cas13 in the dCas13b^{A4}.

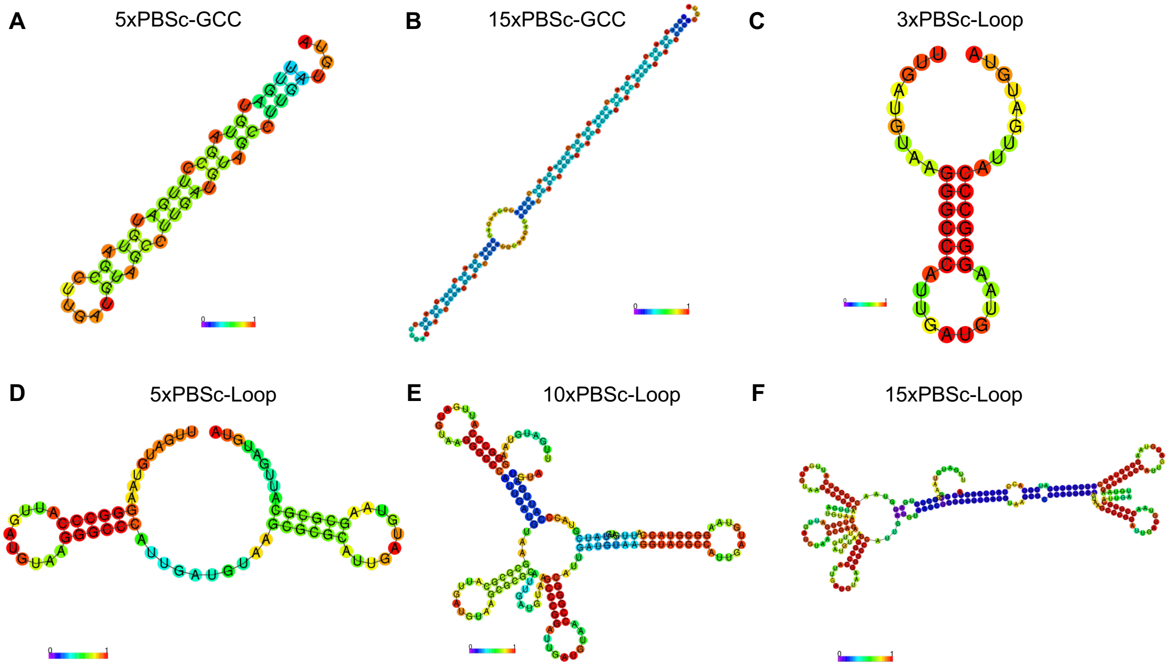

Supplemental figures S2 Predicted secondary structures of PBSc scaffolds by online tool RNAfold. (A-B) 5 and 15 copies of PBSc with GCC linker. The sequence of PBSc is UUGAUGUA. (C-F) Different copies of PBSc stabilized by high GC content stem-loops. 3/5/10/15 copies from C-F separately.

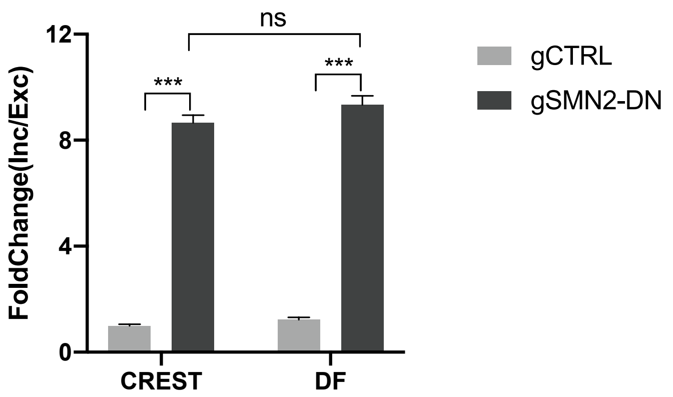

Supplementary figure S3. Comparison of CREST (PUFc-RBFOX1) and direct fusion (DF, dPspCas13b-RBFOX1) on induction of exon inclusion on *SMN2* minigene reporter. For CREST, gRNAs with 10xPBSc with loop were used. All data were displayed as mean ± SD, n = 3. *P<0.05, ** P < 0.01,***P<0.001, ****P<0.0001, ns, not significant, by two-sided t-test.

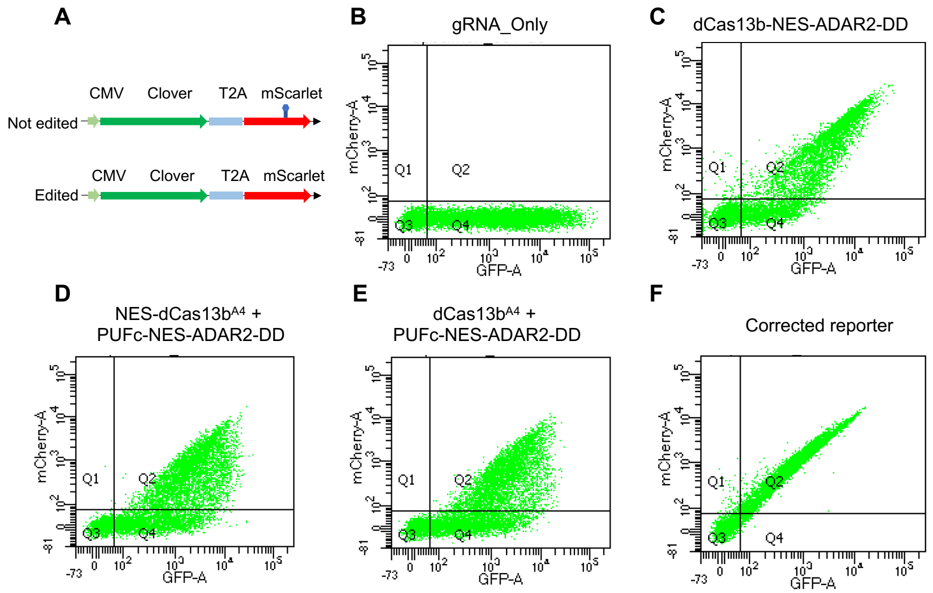

Supplementary figure S4. Representative flow cytometry plots of A-to-G editing by CREST. (A) Diagram of reporter construct for A-to-G editing with a premature stop codon in mScarlet (upper) and the corrected reporter without mutation (lower). (B-E) HEK293T cells were co-transfected with reporter minigene harboring premature stop codon in the coding region of mScarlet and direct fusion or CREST components for correcting this mutation as indicated on the top. (F) HEK293T cells were transfected with reporter minigene without premature stop codon in mScarlet as a positive control.

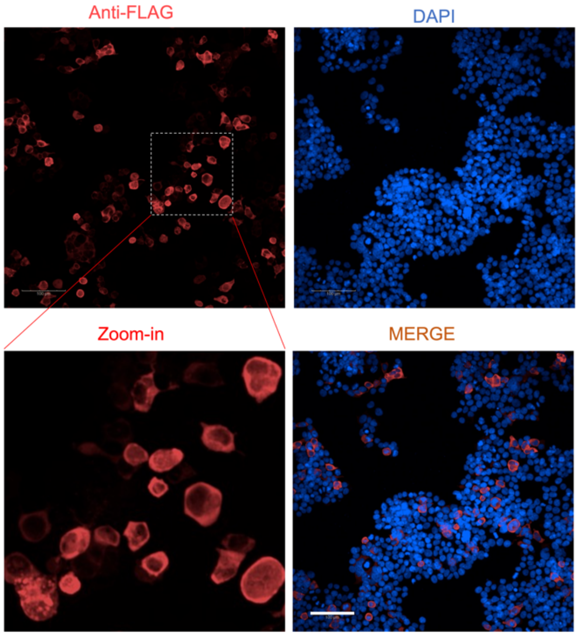

Supplementary figures S5. Sub-cellular localization of dCas13b^{A4} without NES. HEK293T cells were transfected with FLAG-dCas13b^{A4} and stained with anti-FLAG antibody and DAPI, imaged by Phenix. Scale bar: 100 µm.

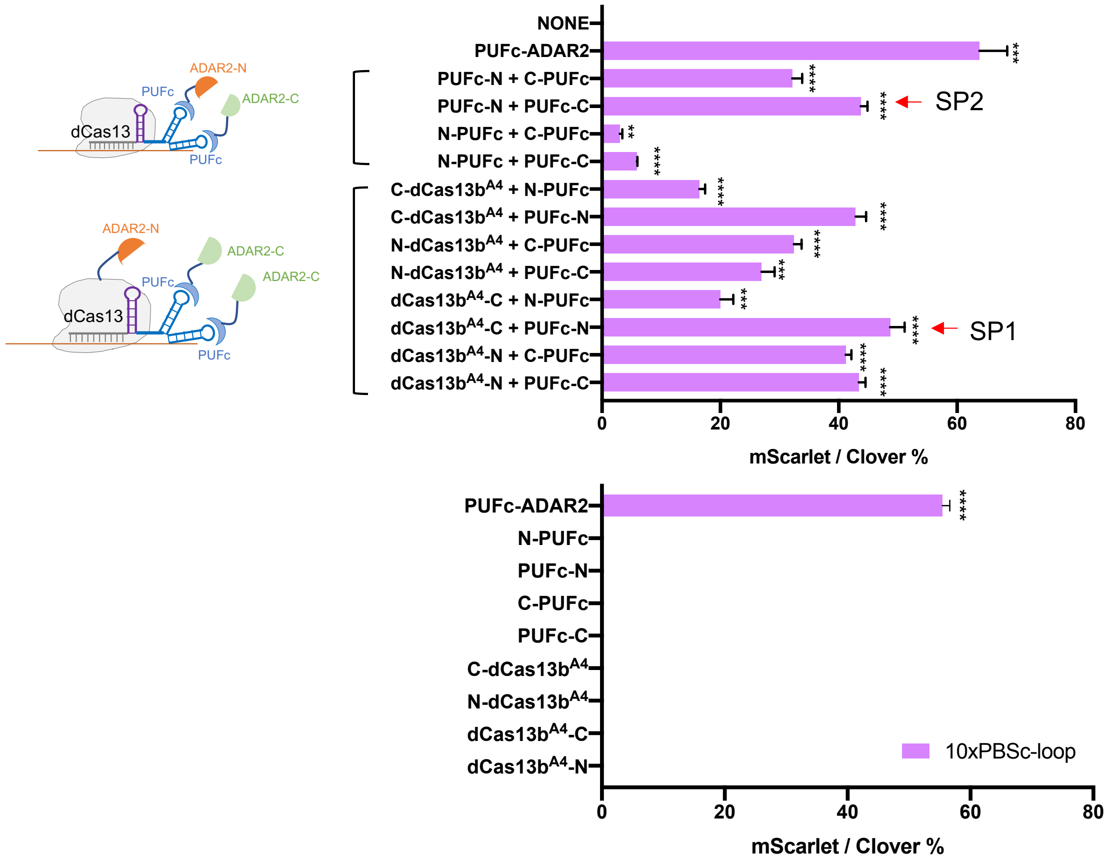

Supplementary figures S6. Half of split ADAR2-DD is insufficient for A-to-G editing. Top: HEK293T cells were transfected with different combinations of split ADAR2-DD and gRNA tagged with 10xPBSc loop. Bottom: HEK293T cells were transfected with either halves of split ADAR2-DD and showed no activity for A-to-G editing. Data were displayed as mean ± S.E.M, n = 3. *P<0.05, ** P < 0.01,***P<0.001, ****P<0.0001, ns, not significant, by two sided t-test.

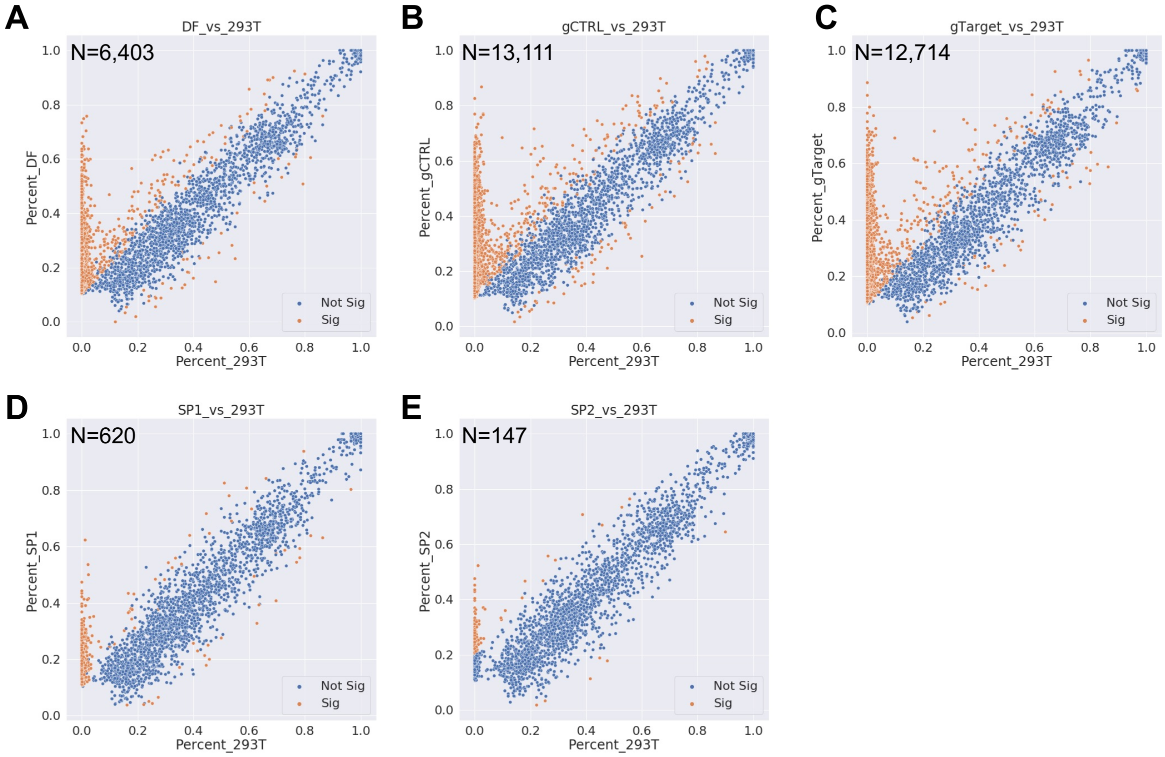

Supplemental figures S7. Transcriptome-wide off-target analysis of A-to-G editing. 2D scatter plot comparing the A-to-G editing yields observed with each construct (y-axis) to the yields observed with the control sample (x-axis, plain 293T). (A) HEK293T cells transfected with direct fusion of dCas13-ADAR2-DD. (B-C) gCtrl and gTarget stand for HEK293T cells co-transfected with dCas13b^{A4}, PUFc-ADAR2-DD and indicated non-targeting control or on-target gRNAs, respectively. (D-E) Reconstitution of base editing activities of ADAR2-DD activity at split sites SP1 or SP2. Dots highlighted in red stand for sites with significant changes in A-to-G editing yields compared to plain 293T without transfection. The number of significant differentially edited sites (N) is listed in each comparison at the top left.

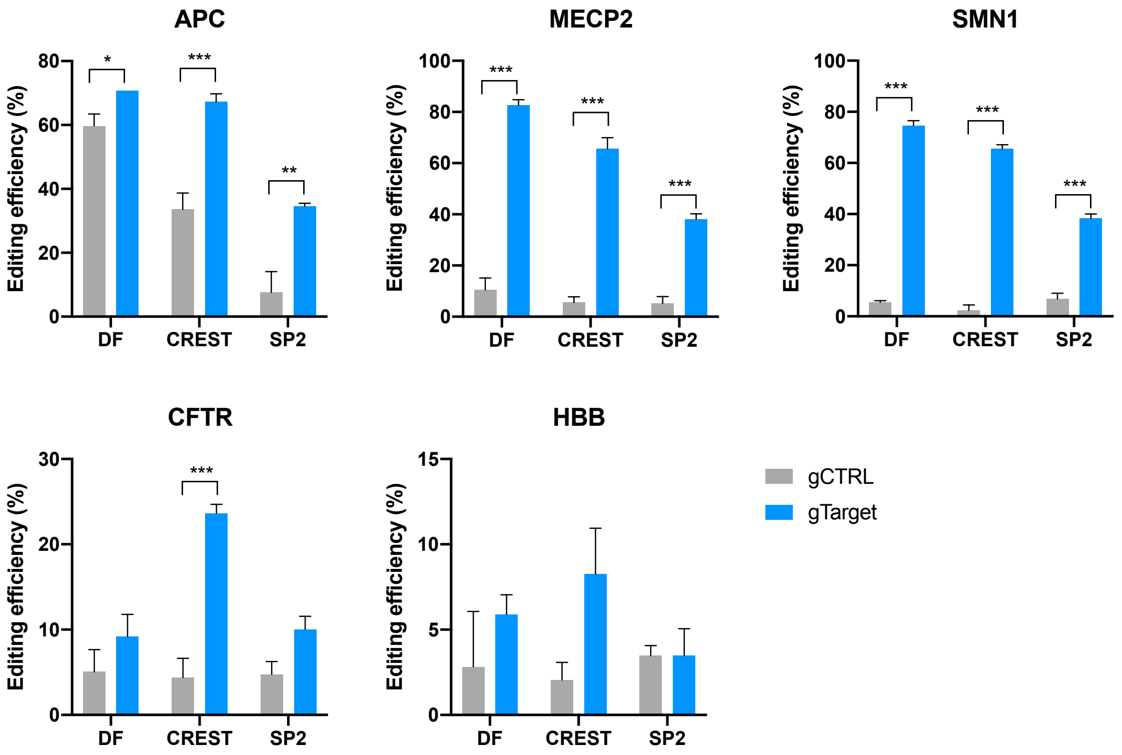

Supplemental figures S8. A-to-G editing of disease-relevant mutations on reporters by CREST system. Editing efficiency was measured by RT-PCR followed by sanger sequencing (Y axis) and different treatments were annotated as X axis. DF: dCas13-ADAR2-DD. CREST: dCas13b^{A4}+PUFc-ADAR2-DD. SP2: dCas13b^{A4}-ADAR2-DD-C + PUFc-ADAR2-DD-N. The target gene name is shown above each graph. Gray columns stand for the non-targeting control gRNA and blue columns stand for on-target gRNAs. All gRNAs were tagged with 3xPBSc and designed with mismatch distance of 22. Data were displayed as mean ± S.E.M, n = 3. *P<0.05, ** P < 0.01,***P<0.001, by two-sided t-test.

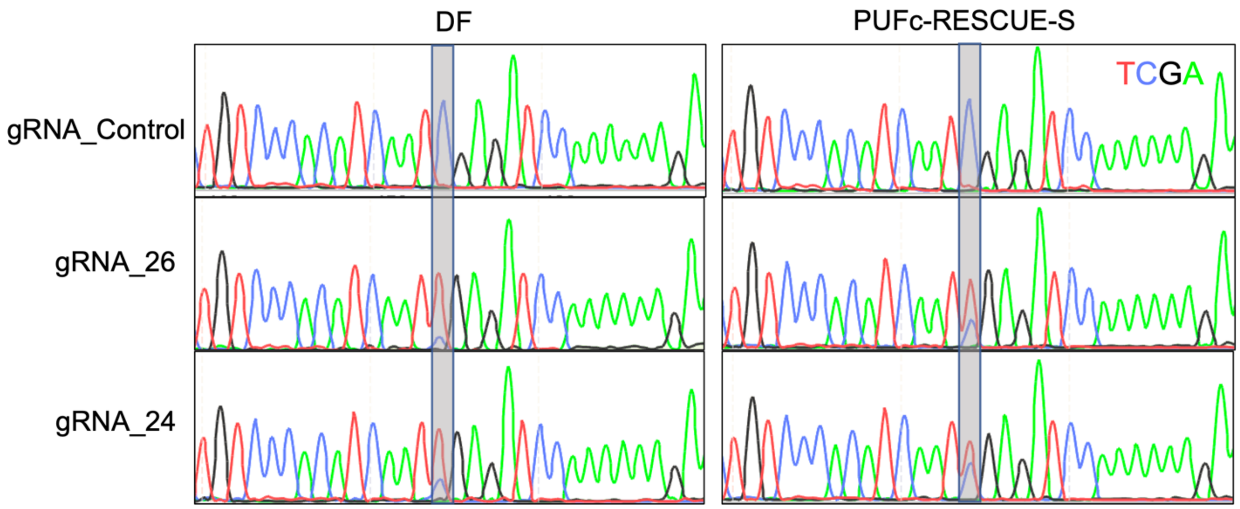

Supplemental figures S9. Representative electropherograms showing Sanger sequencing results of C-to-U base editing. Non-targeting control and on-target gRNA with 24nt and 26nt of mismatch distance were showed. DF stands for dCas13-RESCUE-S. Targeting site was highlighted in gray and colors for different bases were annotated on the top right.

```
ADAR2-DD      QLHLPQVLADAVSRLVLGKFGDLTDNFSSPHARRKVLAGVVMTTGTDVKDAKVISVSTGT
RESCUE-S      QLHLPQVLADAVSRLVIGKFGDLTDNFSSPHARRIGLAGVVMTTGTDVKDAKVICVSTGA
              ****************:*****************  ******************.****:

ADAR2-DD      KCINGEYMSDRGLALNDCHAEIISRRSLLRFLYTQLELYLNNKDDQKRSIFQKSERGGFR
RESCUE-S      KCINGEYLSDRGLALNDCHAEIVSRRSLLRFLYTQLELYLNNEDDQKRSIFQKSERGGFR
              *******:**************:*******************:*****************

ADAR2-DD      LKENVQFHLYISTSPCGDARIFSPHEPILEEPADRHPNRKARGQLRTKIESGQGTIPVRS
RESCUE-S      LKENIQFHLYISTSPCGDARIFSPHEAILEEPADRHPNRKARGQLRTKIEAGQGTIPVRN
              ****:********************* ***********************:********.

ADAR2-DD      NASIQTWDGVLQGERLLTMSCSDKIARWNVVGIQGSLLSIFVEPIYFSSIILGSLYHGDH
RESCUE-S      NASIQTWDGVLQGERLLTMSCSDKIARWNVVGIQGSLLSIFVEPIYFSSIILGSLYHGDH
              ************************************************************

ADAR2-DD      LSRAMYQRISNIEDLPPLYTLNKPLLSGISNAEARQPGKAPNFSVNWTVGDSAIEVINAT
RESCUE-S      LSRAMYQRISNIEDLPPLYTLNKPLLTGISNAEARQPGKAPIFSVNWTVGDSAIEVINAT
              **************************:************** ******************

ADAR2-DD      TGKDELGRASRLCKHALYCRWMRVHGKVPSHLLRSKITKPNVYHESKLAAKEYQAAKARL
RESCUE-S      TGKGELGRASRLCKHALYCRWMRVHGKVPSHLLRSKITKPNVYHETKLAAKEYQAAKARL
              ***.*****************************************:**************

ADAR2-DD      FTAFIKAGLGAWVEKPTEQDQFSLT 385
RESCUE-S      FTAFIKAGLGAWVEKPTEQDQFSLT 385
              *************************
```

Supplemental figures S10. Alignment of ADAR2-DD and RESCUE-S protein sequences. The conserved motif for splitting is highlighted in red and split site is indicated by red arrow between E and P.

QLHLPQVLADAVSRLVIGKFGDLTDNFSSPHARRIG

LAGVVMTTGTDVKDAKVICVSTGAKCINGEYLSDR

GLALNDCHAEIVSRRSLLRFLYTQLELYLNNEDDQK

RSIFQKSERGGFRLKENIQFHLYISTSPCGDARIFSP

1 6

HEAILEEPADRHPNRKARGQLRTKIEAGQGTIPVRN

3 4

NASIQTWDGVLQGERLLTMSCSDKIARWNVVGIQG

2 7

SLLSIFVEPIYFSSIILGSLYHGDHLSRAMYQRISNIE

5

DLPPLYTLNKPLLTGISNAEARQPGKAPIFSVNWTV

GDSAIEVINATTGKGELGRASRLCKHALYCRWMRV

HGKVPSHLLRSKITKPNVYHETKLAAKEYQAAKARL

FTAFIKAGLGAWVEKPTEQDQFSLT

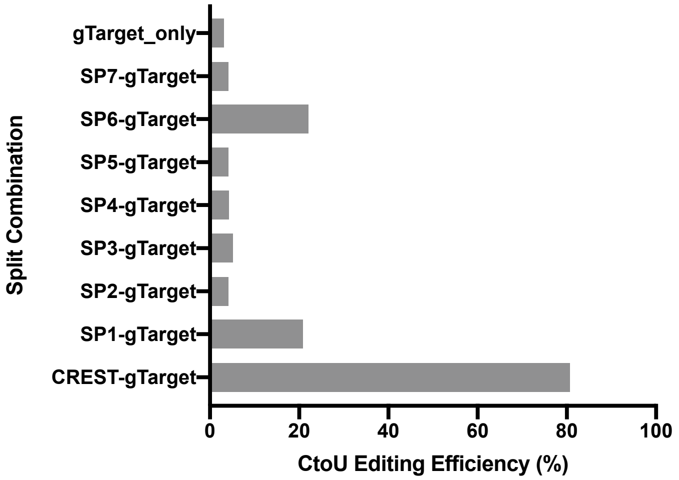

Supplemental figures S11. Optimization of split RESCUE-S. Left: Locations of 7 split sites designed at the low energy regions of RESCUE-S enzyme. Right: Editing efficiency of all split versions in the architecture of dCas13bA4-N/PUFc-C (SP1-gTarget to SP7-gTarget) and full-length enzyme fused to PUFc (CREST-gTarget) quantified by RT-PCR and sanger sequencing. n=1.

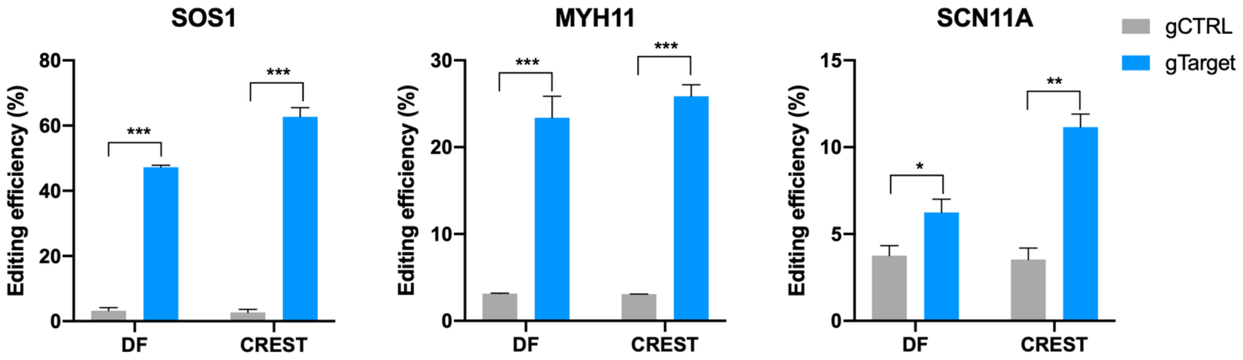

Supplemental figures S12. C-to-U editing of disease-relevant mutations on reporters by CREST system. Editing efficiency was measured by RT-PCR followed sanger sequencing (Y axis) and different treatments are annotated on the X axis. DF: dCas13-ADAR2-DD. CREST: dCas13^{A4}+PUFc-RESCUE-S. The target gene name is shown above each graph. Gray columns stand for the non-targeting control gRNA and blue columns stand for the on-target gRNAs. All gRNAs were tagged with 3xPBSc and designed with mismatch distance of 22. Data were displayed as mean ± SD, n = 3. *P<0.05, ** P < 0.01,***P<0.001, by two-sided t-test.

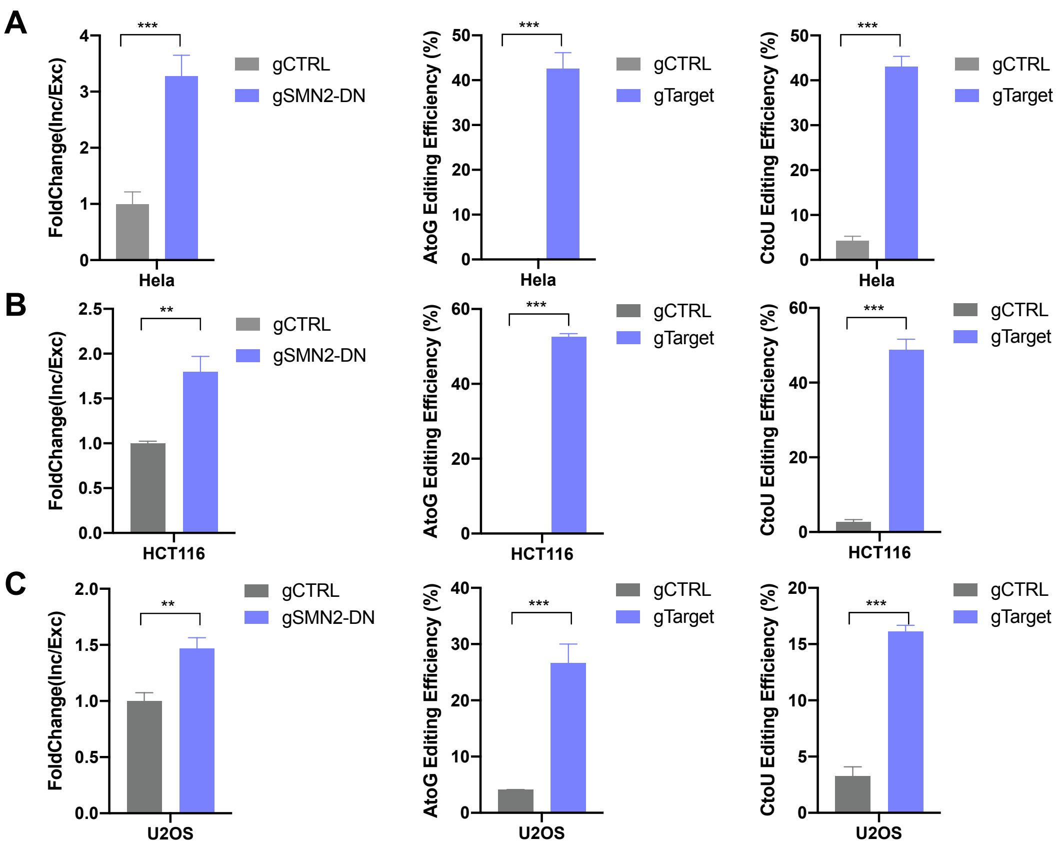

Supplemental figures S13.Validation of CREST in three different cell lines. CREST components (dCas13b^{A4}, PUFc-effectors and gRNA tagged with PBSc) were co-transfected into Hela, HCT116 and U2OS lines as indicated (A-C). Cells were collected 48 hours after transfection for analysis. Induction of splicing was quantified by RT-qPCR (left), and A-to-G / C-to-U editing were quantified by RT-PCR followed by sanger sequencing (Middle and right). Data were displayed as mean ± SD, n = 3. *P<0.05, ** P < 0.01,***P<0.001, by two-sided t-test.

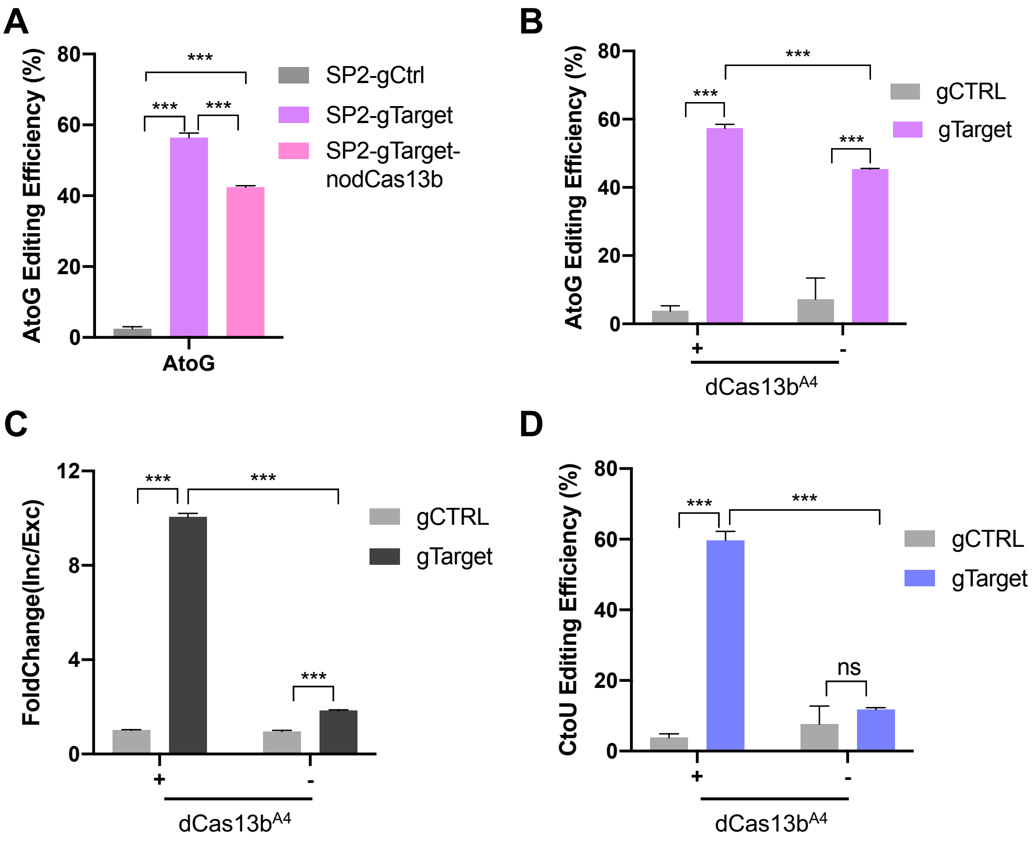

Supplemental figures S14. Requirement of dCas13b^{A4} for different CREST editing modules. (A) HEK293T cells were co-transfected with the core component of SP2 for A-to-G editing (PUFc-N and PUFc-C, gRNA tagged with PBSc) with or without dCas13^{A4} (SP2-gTarget and SP2-gTarget-nodCas13b). (B-D) HEK293T cells were co-transfected with gRNA tagged with PBSc and CREST-PUFc-ADAR2-DD / CREST-PUFc-RBFOX1 / CREST-PUFc-RESCUE-S for A-to-G editing / splicing / C-to-U editing with or without dCas13^{A4} as indicated at the bottom. Induction of splicing was quantified by RT-qPCR (C), and A-to-G / C-to-U editing were quantified by RT-PCR followed by sanger sequencing (B and D). Data were displayed as mean ± SD, n = 3. *P<0.05, ** P < 0.01,***P<0.001, by two-sided t-test.

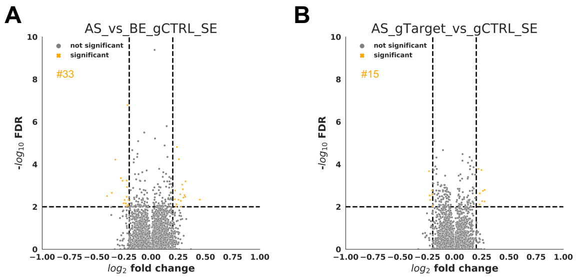

Supplemental figures S15. Off-target analysis of alternative splicing induced by CREST system. Samples from column 1 and 4 in Figure 5 A-C were sent for RNA deep sequencing and subjected to analyze differentially splicing events between groups. (A) Combined all groups transfected with MCP-RBFOX1 and gCTRL (Figure 5A column 1 + 5B column 1) and then compared with HEK293T cells transfected with MCP-ADAR2-DD (AtoG) and PUFc-RESCUE-S (CtoU) and gCTRL (Figure 5C column 1) to evaluate the effect of RBFOX1 itself rather than gRNAs. (B) Combined all groups transfected with MCP-RBFOX1 and compared them based on different gRNAs ([Figure 5A column 4 + 5B column 4] vs [Figure 5A column 1 + 5B column 1.The significant differentially spliced events were filtered by FDR<=0.01 and |change of PSI| > 0.2. Numbers of differentially spliced events are shown at the top left (#) on each graph.

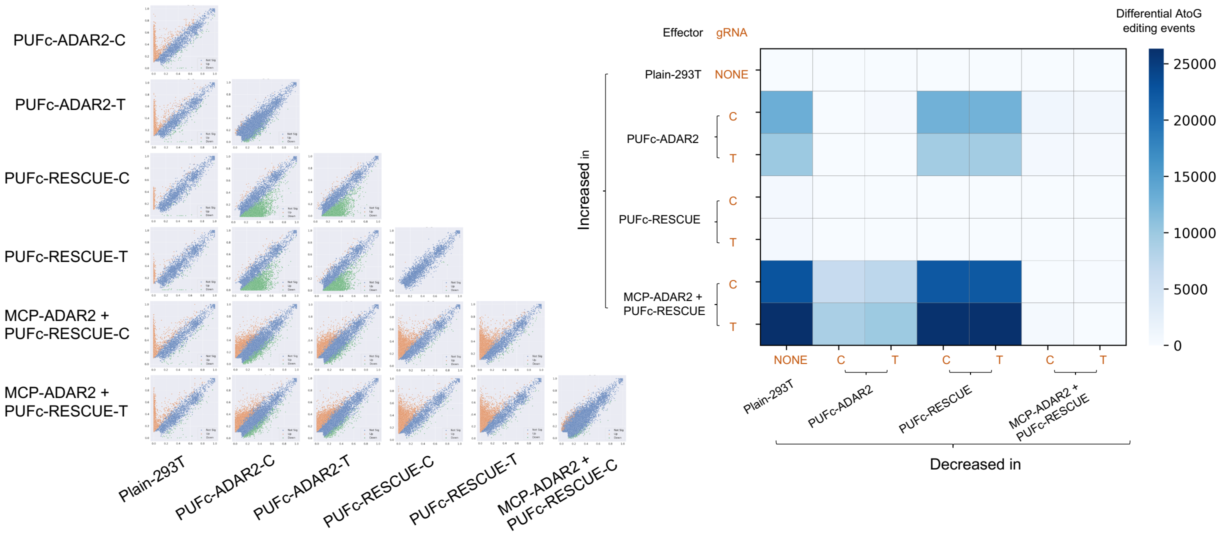

Supplemental figures S16. Off-target events of A-to-G base editing induced by bi-functional CREST system. Samples from column 1 and 4 in Figure 5 A-C were sent for RNA deep sequencing and analyzed for differentially edited events between groups. Left: Scatter plot of A-to-G conversion rates on edited sites across all samples and transfected effectors for each comparison were labeled on x and y axis. C stands for control gRNA and T stands for targeting gRNA. Right: Heatmap to summarize the number of differentially edited events for all pairwise comparisons. Effectors and gRNAs were labeled on both y and x axis. Significant differentially edited events were filter by FDR ≤ 0.05 and absolute difference in editing percentage ≥ 10%.

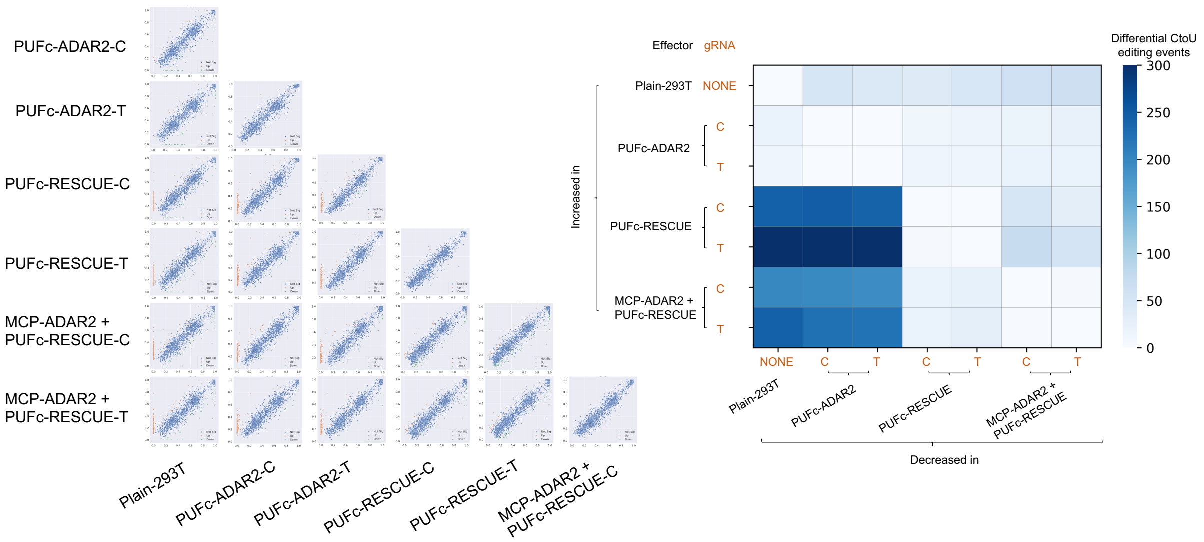

Supplemental figures S17. Off-target events of C-to-U base editing induced by bi-functional CREST system. Samples from column 1 and 4 in Figure 5 A-C were sent for RNA deep sequencing and subjected to analyze differentially edited events between groups. Left: Scatter plot of A-to-G conversion rates on edited sites across all samples and transfected effectors for each comparison were labeled as x and y axis. C stands for control gRNA and T stands for targeting gRNA. Right: Heatmap to summarize the number of differentially edited events for all pairwise comparisons. Effectors and gRNAs were labeled on both y and x axis. Significant differentially edited events were filter by FDR ≤ 0.05 and absolute difference in editing percentage ≥ 10%.

Supplementary Protein Sequences

3xFLAG-ddpspCas13b:

MDYKDHDGDYKDHDIDYKDDDDKIDGGGGSDRRAMNIPALVENQKKYFGTYSVMAMLNAQTVLDHIQKVADIEGEQNENNENLWFHPVMSHLYNAKNGYDKQPEKTMFIIERLQSYFPFLKIMAENQREYSNGKYKQNRVEVNSNDIFEVLKRAFGVLKMYRDLTNAYKTYEEKLNDGCEFLTSTEQPLSGMINNYYTVALRNMNERYGYKTEDLAFIQDKRFKFVKDAYGKKKSQVNTGFFLSLQDYNGDTQKKLHLSGVGIALLICLFLDKQYINIFLSRLPIFSSYNAQSEERRIIIRSFGINSIKLPKDRIHSEKSNKSVAMDMLNEVKRCPDELFTTLSAEKQSRFRIISDDHNEVLMKRSSDRFVPLLLQYIDYGKLFDHIRFHVNMGKLRYLLAAAATCIDGQTRVRVIEQPLNGFGRLEEAETMRKQENGTFGNSGIRIRDFENMKRDDANPANYPYIVDTYTHYILENNKVEMFINDKEDSAPLLPVIEDDRYVVKTIPSCRMSTLEIPAMAFHMFLFGSKKTEKLIVDVHNRYKRLFQAMQKEEVTAENIASFGIAESDLPQKILDLISGNAHGKDVDAFIRLTVDDMLTDTERRIKRFKDDRKSIRSADNKMGKRGFKQISTGKLADFLAKDIVLFQPSVNDGENKITGLNYRIMQSAIAVYDSGDDYEAKQQFKLMFEKARLIGKGTTEPHPFLYKVFARSIPANAVEFYERYLIERKFYLTGLSNEIKKGNRVDVPFIRRDQNKWKTPAMKTLGRIYSEDLPVELPRQMFDNEIKSHLKSLPQMEGIDFNNANVTYLIAEYMKRVLDDDFQTFYQWNRNYRYMDMLKGEYDRKGSLQHCFTSVEEREGLWKERASRTERYRKQASNKIRSNRQMRNASSEEIETILDKRLSNSRNEYQKSEKVIRRYRVQDALLFLLAKKTLTELADFDGERFKLKEIMPDAEKGILSEIMPMSFTFEKGGKKYTITSEGMKLKNYGDFFVLASDKRIGNLLELVGSDIVSKEDIMEEFNKYDQCRPEISSIVFNLEKWAFDTYPELSARVDREEKVDFKSILKILLNNKNINKEQSDILRKIRNAFDANNYPDKGVVEIKALPEIAMSIKKAFGEYAIMKGGRGGGGSGGGGSGGGGSGPA

hcoRBFOX1N-MCP-RBFOX1C

MNCEREQLRGNQEAAAAPDTMAQPYASAQFAPPQNGIPAEYTAPHPHPAPEYTGQTTVPEHTLNLYPPAQTHSEQSPADTSAQTVSGTATQTDDAAPTDGQPQTQPSENTENKSQPKGGGGSGRAMASNFTQFVLVDNGGTGDVTVAPSNFANGVAEWISSNSRSQAYKVTCSVRQSSAQKRKYTIKVEVPKVATQTVGGVELPVAAWRSYLNMELTIPIFATNSDCELIVKAMQGLLKDGNPIPSAIAANSGIYSAGGRGGGGSGGGGSGGGGSGPANATARVMTNKKTVNPYTNGWKLNPVVGAVYSPEFYAGTVLLCQANQEGSSMYSAPSSLVYTSAMPGFPYPAATAAAAYRGAHLRGRGRTVYNTFRAAAPPPPIPAYGGVVYQDGFYGADIYGGYAAYRYAQPTPATAAAYSDSYGRVYAADPYHHALAPAPTYGVGAMNAFAPLTDAKTRSHADDVGLVLSSLQASIYRGGYNRFAPY

hcoRBFOX1N-PUFc-2xNLS-RBFOX1C

MNCEREQLRGNQEAAAAPDTMAQPYASAQFAPPQNGIPAEYTAPHPHPAPEYTGQTTVPEHTLNLYPPAQTHSEQSPADTSAQTVSGTATQTDDAAPTDGQPQTQPSENTENKSQPKGGGGSGRAGILPPKKKRKVSRGRSRLLEDFRNNRYPNLQLREIAGHIMEFSQDQHGSRFIQLKLERATPAERQLVFNEILQAAYQLMVDVFGNYVIQKFFEFGSLEQKLALAERIRGHVLSLALQMYGSRVIEKALEFIPSDQQNEMVRELDGHVLKCVKDQNGNHVVQKCIECVQPQSLQFIIDAFKGQVFALSTHPYGCRVIQRILEHCLPDQTLPILEELHQHTEQLVQDQYGSYVIEHVLEHGRPEDKSKIVAEIRGNVLVLSQHKFANNVVQKCVTHASRTERAVLIDEVCTMNDGPHSALYTMMKDQYANYVVQKMIDVAEPGQRKIVMHKIRPHIATLRKYTYGKHILAKLEKYYMKNGVDLGDPKKKRKVDPKKKRKVGGRGGGGSGGGGSGGGGSGPANATARVMTNKKTVNPYTNGWKLNPVVGAVYSPEFYAGTVLLCQANQEGSSMYSAPSSLVYTSAMPGFPYPAATAAAAYRGAHLRGRGRTVYNTFRAAAPPPPIPAYGGVVYQDGFYGADIYGGYAAYRYAQPTPATAAAYSDSYGRVYAADPYHHALAPAPTYGVGAMNAFAPLTDAKTRSHADDVGLVLSSLQASIYRGGYNRFAPY

MCP-NES-ADAR2DD(E448Q)

MASNFTQFVLVDNGGTGDVTVAPSNFANGVAEWISSNSRSQAYKVTCSVRQSSAQKRKYTIKVEVPKVATQTVGGVELPVAAWRSYLNMELTIPIFATNSDCELIVKAMQGLLKDGNPIPSAIAANSGIYSAGGRGGGGSGGGGSGGGGSGPALQLPPLERLTLGSGGGGSQLHLPQVLADAVSRLVLGKFGDLTDNFSSPHARRKVLAGVVMTTGTDVKDAKVISVSTGTKCINGEYMSDRGLALNDCHAEIISRRSLLRFLYTQLELYLNNKDDQKRSIFQKSERGGFRLKENVQFHLYISTSPCGDARIFSPHEPILEEPADRHPNRKARGQLRTKIESGQGTIPVRSNASIQTWDGVLQGERLLTMSCSDKIARWNVVGIQGSLLSIFVEPIYFSSIILGSLYHGDHLSRAMYQRISNIEDLPPLYTLNKPLLSGISNAEARQPGKAPNFSVNWTVGDSAIEVINATTGKDELGRASRLCKHALYCRWMRVHGKVPSHLLRSKITKPNVYHESKLAAKEYQAAKARLFTAFIKAGLGAWVEKPTEQDQFSLTNV

PUFc-NES-ADAR2DD(E488Q)

MNVGGGGSGGGGSGGGGSGRASRGRSRLLEDFRNNRYPNLQLREIAGHIMEFSQDQHGSRFIQLKLERATPAERQLVFNEILQAAYQLMVDVFGNYVIQKFFEFGSLEQKLALAERIRGHVLSLALQMYGSRVIEKAL

EFIPSDQQNEMVRELDGHVLKCVKDQNGNHVVQKCIECVQPQSLQFIIDAFKGQVFALSTHPYGCRVIQR
ILEHCLPDQTLPILEELHQHTEQLVQDQYGSYVIEHVLEHGRPEDKSKIVAEIRGNVLVLSQHKFANNVVQ
KCVTHASRTERAVLIDEVCTMNDGPHSALYTMMKDQYANYVVQKMIDVAEPGQRKIVMHKIRPHIATLRK
YTYGKHILAKLEKYYMKNGVDLGGGRGGGGSGGGGSGGGGSGPALQLPPLERLTLGSGGGGSQLHLP
QVLADAVSRLVLGKFGDLTDNFSSPHARRKVLAGVVMTTGTDVKDAKVISVSTGTKCINGEYMSDRGLAL
NDCHAEIISRRSLLRFLYTQLELYLNNKDDQKRSIFQKSERGGFRLKENVQFHLYISTSPCGDARIFSPHEP
ILEEPADRHPNRKARGQLRTKIESGQGTIPVRSNASIQTWDGVLQGERLLTMSCSDKIARWNVVGIQGSL
LSIFVEPIYFSSIILGSLYHGDHLSRAMYQRISNIEDLPPLYTLNKPLLSGISNAEARQPGKAPNFSVNWTVG
DSAIEVINATTGKDELGRASRLCKHALYCRWMRVHGKVPSHLLRSKITKPNVYHESKLAAKEYQAAKARL
FTAFIKAGLGAWVEKPTEQDQFSLTNV

PUFc-NES-RESCUE-S
MNVGGGGSGGGGSGGGGSGRASRGRSRLLEDFRNNRYPNLQLREIAGHIMEFSQDQHGSRFIQLKLE
RATPAERQLVFNEILQAAYQLMVDVFGNYVIQKFFEFGSLEQKLALAERIRGHVLSLALQMYGSRVIEKAL
EFIPSDQQNEMVRELDGHVLKCVKDQNGNHVVQKCIECVQPQSLQFIIDAFKGQVFALSTHPYGCRVIQR
ILEHCLPDQTLPILEELHQHTEQLVQDQYGSYVIEHVLEHGRPEDKSKIVAEIRGNVLVLSQHKFANNVVQ
KCVTHASRTERAVLIDEVCTMNDGPHSALYTMMKDQYANYVVQKMIDVAEPGQRKIVMHKIRPHIATLRK
YTYGKHILAKLEKYYMKNGVDLGGGRGGGGSGGGGSGGGGSGPALQKKLEELELGSQLHLPQVLADAV
SRLVIGKFGDLTDNFSSPHARRIGLAGVVMTTGTDVKDAKVICVSTGAKCINGEYLSDRGLALNDCHAEIV
SRRSLLRFLYTQLELYLNNEDDQKRSIFQKSERGGFRLKENIQFHLYISTSPCGDARIFSPHEAILEEPADR
HPNRKARGQLRTKIEAGQGTIPVRNNASIQTWDGVLQGERLLTMSCSDKIARWNVVGIQGSLLSIFVEPIY
FSSIILGSLYHGDHLSRAMYQRISNIEDLPPLYTLNKPLLTGISNAEARQPGKAPIFSVNWTVGDSAIEVINA
TTGKGELGRASRLCKHALYCRWMRVHGKVPSHLLRSKITKPNVYHETKLAAKEYQAAKARLFTAFIKAGL
GAWVEKPTEQDQFSLTVDGSGSGSLPPLERLTL

NES-PUFc-ADAR2DD(E488Q)-splitN
MLPPLERLTLGGGGSGRASRGRSRLLEDFRNNRYPNLQLREIAGHIMEFSQDQHGSRFIQLKLERATPAE
RQLVFNEILQAAYQLMVDVFGNYVIQKFFEFGSLEQKLALAERIRGHVLSLALQMYGSRVIEKALEFIPSDQ
QNEMVRELDGHVLKCVKDQNGNHVVQKCIECVQPQSLQFIIDAFKGQVFALSTHPYGCRVIQRILEHCLP
DQTLPILEELHQHTEQLVQDQYGSYVIEHVLEHGRPEDKSKIVAEIRGNVLVLSQHKFANNVVQKCVTHAS
RTERAVLIDEVCTMNDGPHSALYTMMKDQYANYVVQKMIDVAEPGQRKIVMHKIRPHIATLRKYTYGKHIL
AKLEKYYMKNGVDLGGGRGGGGSGGGGSGGGGSGPAQLHLPQVLADAVSRLVLGKFGDLTDNFSSPH
ARRKVLAGVVMTTGTDVKDAKVISVSTGTKCINGEYMSDRGLALNDCHAEIISRRSLLRFLYTQLELYLNN
KDDQKRSIFQKSERGGFRLKENVQFHLYISTSPCGDARIFSPHEPILEE

NES-PUFc-ADAR2DD(E488Q)-splitC
MLPPLERLTLGGGGSGRASRGRSRLLEDFRNNRYPNLQLREIAGHIMEFSQDQHGSRFIQLKLERATPAE
RQLVFNEILQAAYQLMVDVFGNYVIQKFFEFGSLEQKLALAERIRGHVLSLALQMYGSRVIEKALEFIPSDQ
QNEMVRELDGHVLKCVKDQNGNHVVQKCIECVQPQSLQFIIDAFKGQVFALSTHPYGCRVIQRILEHCLP
DQTLPILEELHQHTEQLVQDQYGSYVIEHVLEHGRPEDKSKIVAEIRGNVLVLSQHKFANNVVQKCVTHAS
RTERAVLIDEVCTMNDGPHSALYTMMKDQYANYVVQKMIDVAEPGQRKIVMHKIRPHIATLRKYTYGKHIL
AKLEKYYMKNGVDLGGGRGGGGSGGGGSGGGGSGPAPADRHPNRKARGQLRTKIESGQGTIPVRSNA
SIQTWDGVLQGERLLTMSCSDKIARWNVVGIQGSLLSIFVEPIYFSSIILGSLYHGDHLSRAMYQRISNIEDL
PPLYTLNKPLLSGISNAEARQPGKAPNFSVNWTVGDSAIEVINATTGKDELGRASRLCKHALYCRWMRVH
GKVPSHLLRSKITKPNVYHESKLAAKEYQAAKARLFTAFIKAGLGAWVEKPTEQDQFSLTNV

NES-ddPspCas13b-ARA2DD(E488Q)-SplitC
MLPPLERLTLGGGGSGRAMNIPALVENQKKYFGTYSVMAMLNAQTVLDHIQKVADIEGEQNENNENLWF
HPVMSHLYNAKNGYDKQPEKTMFIIERLQSYFPFLKIMAENQREYSNGKYKQNRVEVNSNDIFEVLKRAF
GVLKMYRDLTNAYKTYEEKLNDGCEFLTSTEQPLSGMINNYYTVALRNMNERYGYKTEDLAFIQDKRFKF
VKDAYGKKKSQVNTGFFLSLQDYNGDTQKKLHLSGVGIALLICLFLDKQYINIFLSRLPIFSSYNAQSEERRI
IIRSFGINSIKLPKDRIHSEKSNKSVAMDMLNEVKRCPDELFTTLSAEKQSRFRIISDDHNEVLMKRSSDRF
VPLLLQYIDYGKLFDHIRFHVNMGKLRYLLAAAATCIDGQTRVRVIEQPLNGFGRLEEAETMRKQENGTFG
NSGIRIRDFENMKRDDANPANYPYIVDTYTHYILENNKVEMFINDKEDSAPLLPVIEDDRYVVKTIPSCRMS
TLEIPAMAFHMFLFGSKKTEKLIVDVHNRYKRLFQAMQKEEVTAENIASFGIAESDLPQKILDLISGNAHGK
DVDAFIRLTVDDMLTDTERRIKRFKDDRKSIRSADNKMGKRGFKQISTGKLADFLAKDIVLFQPSVNDGEN

KITGLNYRIMQSAIAVYDSGDDYEAKQQFKLMFEKARLIGKGTTEPHPFLYKVFARSIPANAVEFYERYLIE
RKFYLTGLSNEIKKGNRVDVPFIRRDQNKWKTPAMKTLGRIYSEDLPVELPRQMFDNEIKSHLKSLPQME
GIDFNNANVTYLIAEYMKRVLDDDFQTFYQWNRNYRYMDMLKGEYDRKGSLQHCFTSVEEREGLWKER
ASRTERYRKQASNKIRSNRQMRNASSEEIETILDKRLSNSRNEYQKSEKVIRRYRVQDALLFLLAKKTLTE
LADFDGERFKLKEIMPDAEKGILSEIMPMSFTFEKGGKKYTITSEGMKLKNYGDFFVLASDKRIGNLLELVG
SDIVSKEDIMEEFNKYDQCRPEISSIVFNLEKWAFDTYPELSARVDREEKVDFKSILKILLNNKNINKEQSDI
LRKIRNAFDANNYPDKGVVEIKALPEIAMSIKKAFGEYAIMKGGRGGGGSGGGGSGGGGSGPAPADRHP
NRKARGQLRTKIESGQGTIPVRSNASIQTWDGVLQGERLLTMSCSDKIARWNVVGIQGSLLSIFVEPIYFS
SIILGSLYHGDHLSRAMYQRISNIEDLPPLYTLNKPLLSGISNAEARQPGKAPNFSVNWTVGDSAIEVINATT
GKDELGRASRLCKHALYCRWMRVHGKVPSHLLRSKITKPNVYHESKLAAKEYQAAKARLFTAFIKAGLGA
WVEKPTEQDQFSLTNV

NES-ddPspCas13b-RESCUE-S-splitN
MLPPLERLTLGGGGSGRAMNIPALVENQKKYFGTYSVMAMLNAQTVLDHIQKVADIEGEQNENNENLWF
HPVMSHLYNAKNGYDKQPEKTMFIIERLQSYFPFLKIMAENQREYSNGKYKQNRVEVNSNDIFEVLKRAF
GVLKMYRDLTNAYKTYEEKLNDGCEFLTSTEQPLSGMINNYYTVALRNMNERYGYKTEDLAFIQDKRFKF
VKDAYGKKKSQVNTGFFLSLQDYNGDTQKKLHLSGVGIALLICLFLDKQYINIFLSRLPIFSSYNAQSEERRI
IIRSFGINSIKLPKDRIHSEKSNKSVAMDMLNEVKRCPDELFTTLSAEKQSRFRIISDDHNEVLMKRSSDRF
VPLLLQYIDYGKLFDHIRFHVNMGKLRYLLAAAATCIDGQTRVRVIEQPLNGFGRLEEAETMRKQENGTFG
NSGIRIRDFENMKRDDANPANYPYIVDTYTHYILENNKVEMFINDKEDSAPLLPVIEDDRYVVKTIPSCRMS
TLEIPAMAFHMFLFGSKKTEKLIVDVHNRYKRLFQAMQKEEVTAENIASFGIAESDLPQKILDLISGNAHGK
DVDAFIRLTVDDMLTDTERRIKRFKDDRKSIRSADNKMGKRGFKQISTGKLADFLAKDIVLFQPSVNDGEN
KITGLNYRIMQSAIAVYDSGDDYEAKQQFKLMFEKARLIGKGTTEPHPFLYKVFARSIPANAVEFYERYLIE
RKFYLTGLSNEIKKGNRVDVPFIRRDQNKWKTPAMKTLGRIYSEDLPVELPRQMFDNEIKSHLKSLPQME
GIDFNNANVTYLIAEYMKRVLDDDFQTFYQWNRNYRYMDMLKGEYDRKGSLQHCFTSVEEREGLWKER
ASRTERYRKQASNKIRSNRQMRNASSEEIETILDKRLSNSRNEYQKSEKVIRRYRVQDALLFLLAKKTLTE
LADFDGERFKLKEIMPDAEKGILSEIMPMSFTFEKGGKKYTITSEGMKLKNYGDFFVLASDKRIGNLLELVG
SDIVSKEDIMEEFNKYDQCRPEISSIVFNLEKWAFDTYPELSARVDREEKVDFKSILKILLNNKNINKEQSDI
LRKIRNAFDANNYPDKGVVEIKALPEIAMSIKKAFGEYAIMKGGRGGGGSGGGGSGGGGSGPAQLHLPQ
VLADAVSRLVIGKFGDLTDNFSSPHARRIGLAGVVMTTGTDVKDAKVICVSTGAKCINGEYLSDRGLALND
CHAEIVSRRSLLRFLYTQLELYLNNEDDQKRSIFQKSERGGFRLKENIQFHLYISTSPCGDARIFSPHEAILE
E

NES-PUFc-RESCUE-S-splitC
MLPPLERLTLGGGGSGRASRGRSRLLEDFRNNRYPNLQLREIAGHIMEFSQDQHGSRFIQLKLERATPAE
RQLVFNEILQAAYQLMVDVFGNYVIQKFFEFGSLEQKLALAERIRGHVLSLALQMYGSRVIEKALEFIPSDQ
QNEMVRELDGHVLKCVKDQNGNHVVQKCIECVQPQSLQFIIDAFKGQVFALSTHPYGCRVIQRILEHCLP
DQTLPILEELHQHTEQLVQDQYGSYVIEHVLEHGRPEDKSKIVAEIRGNVLVLSQHKFANNVVQKCVTHAS
RTERAVLIDEVCTMNDGPHSALYTMMKDQYANYVVQKMIDVAEPGQRKIVMHKIRPHIATLRKYTYGKHIL
AKLEKYYMKNGVDLGGGRGGGGSGGGGSGGGGSGPAPADRHPNRKARGQLRTKIEAGQGTIPVRNNA
SIQTWDGVLQGERLLTMSCSDKIARWNVVGIQGSLLSIFVEPIYFSSIILGSLYHGDHLSRAMYQRISNIEDL
PPLYTLNKPLLTGISNAEARQPGKAPIFSVNWTVGDSAIEVINATTGKGELGRASRLCKHALYCRWMRVH
GKVPSHLLRSKITKPNVYHETKLAAKEYQAAKARLFTAFIKAGLGAWVEKPTEQDQFSLT

Supplementary Table S1

PCR Primers	
AtoG reporter editing Forward	GGCTCCGAATTCACCGGTG
AtoG reporter editing Reverse	CTTCTTCTGCATTACGGGGC
CtoU reporter editing Forward	GTGGGAGCGCGTGATGAACT
CtoU reporter editing Reverse	AAAGCTGGGTCGAATTCTTAATTAA
KRAS Forward	CCAGGCCTGCTGAAAATGAC
KRAS Reverse	GAAGGCATCATCAACACCCAGA
Sanger sequencing primer	
AtoG reporter sequencing	GCCCGACAACCACTACCTGA
CtoU reporter sequencing	TGGACATCACCTCCCACAACG
KRAD sequencing	CTGGTCCCTCATTGCACTG

qRT-PCR primers	
SMN2-Forward	GCTCTTAAGGCTAGAGTACTTAATACGA
SMN2-Inclusion-Reverse	CTTCTTTTTGATTTTGTCTAAAACCCATATAATAG
SMN2-Exclusion-Reverse	CTCTATGCCAGCATTTCCATATAATAG
KIF21A-Forward	TGGAAGGTCGACTCAAACAA
KIF21A-Reverse	TGGGCTGTTTAAAGGAGCAT

gRNA spacer sequence oligos	
Ctrl	ACTCAAAAGGAAGTGACAAGAA
SMN1-gRNA1	GTAAGATTCACTTTCATAATGC
SMN1-gRNA2	GTAGGGATGTAGATTAACCTTT
SMN1-gRNA3	GCTGGTCTGCCTACTAGTGATA
mScarlet-A-to-G-2	GACATGAACTGAGGGGACAGGATGTCCCA
mScarlet-A-to-G-4	GATGAACTGAGGGGACAGGATGTCCCAGG
mScarlet-A-to-G-6	GATGAACTGAGGGGACAGGATGTCCCAGGAG
mScarlet-A-to-G-8	GAACTGAGGGGACAGGATGTCCCAGGAGAA
mScarlet-A-to-G-10	GACTGAGGGGACAGGATGTCCCAGGAGAAGG
mScarlet-A-to-G-12	GTGAGGGGACAGGATGTCCCAGGAGAAGGGC
mScarlet-A-to-G-14	GAGGGGACAGGATGTCCCAGGAGAAGGGCAG
mScarlet-A-to-G-16	GGGACAGGATGTCCCAGGAGAAGGGCAGGG
mScarlet-A-to-G-18	GACAGGATGTCCCAGGAGAAGGGCAGGGGG
mScarlet-A-to-G-20	GCAGGATGTCCCAGGAGAAGGGCAGGGGGCC
mScarlet-A-to-G-22	GGATGTCCCAGGAGAAGGGCAGGGGGCCAC
mScarlet-A-to-G-24	GATGTCCCAGGAGAAGGGCAGGGGGCCACCC

mScarlet-A-to-G-26	GTCCCAGGAGAAGGGCAGGGGGCCACCCTT
mScarlet-A-to-G-28	GCCCAGGAGAAGGGCAGGGGGCCACCCTTGG
mScarlet-A-to-G-30	GCAGGAGAAGGGCAGGGGGCCACCCTTGGTC
mScarlet-C-to-U-20	CTGCCTGTCCCACATCAATGGAGATCCAAAC
mScarlet-C-to-U-22	GTGGATCTCCATTGATGTGGGACAGGCAGAT
mScarlet-C-to-U-24	GATCTCCATTGATGTGGGACAGGCAGATCA
mScarlet-C-to-U-26	GTCTCCATTGATGTGGGACAGGCAGATCAGG
mScarlet-C-to-U-28	GTCCATTGATGTGGGACAGGCAGATCAGGGC
APC	GCCACTCCCAACAGGTTTCACAGTAAGCGC
MECP2	GTCCGTGTCCAGCCTTCAGGCAGGGTGGGGT
SMN1	GCTTCTGACCAAATGGCAGAACATTTGTCCC
CFTR	GCTTTCCTCCACTGTTGCAAAGTTATTGAA
HBB	gCTCTGGGTCCAAGGGTAGACCACCAGCAGC
SOS1	gCATCTGTCCTTTCTACTGTATCTTCTATAT
MYH11	gTGGACTGCCGCTCCTGCACCTGCGCCTCCA
SCN11A	gCAACAGCCCGGGTTAAGTTAATCAGGTAGA
KIF21A-gRNA1	gACAATTAGTAATTCATGCAGCTG
KIF21A-gRNA2	gTGCAAAAACCACTTGACCGCCAA
KIF21A-gRNA3	GACTTAGTGTGTTTGTGGGCATG
KRAS-AtoG-editing	GTTTCTCCATCAATTACCACTTGCTTCCTGTAGGAATCCTCTATTGTTGGA
KRAS-CtoU-editing	gATTCCTCCACAAAATGATTCTGAATTAGCT

RNA scaffold sequence	
1xMS2	CGTACACCATCAGGGTACG
2xMS2	CGTACACCATCAGGGTACGCagatGCGTACACCATCAGGGTACG
1xPBSc	ttgatgta
2xPBSc	ttgatgtagccttgatgta
3xPBSc-Loop	ttgatgtaAGGGCCCAttgatgtaAGGGCCCAttgatgta
5xPBSc-Loop	ttgatgtaAGGGCCCAttgatgtaAGGGCCCAttgatgtaAGCGCGCAttgatgtaAGCGCGCAttgatgta
10xPBSc-Loop	ttgatgtaAGGGCCCAttgatgtaAGGGCCCAttgatgtaAGCGCGCAttgatgtaAGCGCGCAttgatgtaAGCCCGGAttgatgtaACCGGGCAttgatgtaAGGTACGCCAttgatgtaAGGCGTACCAttgatgtaTCGTACCCAttgatgta
15xPBSc-Loop	ttgatgtaaGGCCGCTttgatgtaACGCGGTCAttgatgtaAGACCGCGAttgatgtaACTCGGAttgatgtaACCGAGATTtgatgtaAGGGCCCAttgatgtaAGGGCCCAttgatgta

	AGCGCGCAttgatgtaAGCGCGCAttgatgtaAGCCCGGAttgatgtaACCGGGCAttgatgtaAGGTACGCCAttgatgtaAGGCGTACCAttgatgtaTCGTACCCAttgatgta
5xPBSc-GCC	ttgatgtagccttgatgtagccttgatgtagccttgatgtagccttgatgta
15xPBSc-GCC	ttgatgtagccttgatgtagccttgatgtagccttgatgtagccttgatgtaagatttgatgtagccttgatgtagccttgatgtagccttgatgtagccttgatgtaagatttgatgtagccttgatgtagccttgatgtagccttgatgtagccttgatgta

Supplementary Table S2

Plasmid	Description/Experimental Purpose	ID
pCR8-PspCas13b_gRNA[ccdbCam]_1xMS2b	backbone for gRNA cloning of dpspCas13b tagged by 1xMS2	196847
pCR8-PspCas13b_gRNA[ccdbCam]-2xPP7	backbone for gRNA cloning of dpspCas13b tagged by 2xPP7	196846
gzk326-dpspCas13b_gRNA[ccdbCam]-5xPBSc_Loop	backbone for gRNA cloning of dpspCas13b tagged by 5xPBSc_Loop	196845
gzk328-dpspCas13b_gRNA[ccdbCam]-10xPBSc_Loop	backbone for gRNA cloning of dpspCas13b tagged by 10xPBSc_Loop	196844
pzk490-pmax-NES-dpspCas13b(AAAA)-FseI[ADAR2-DD-splitC]	Express NES-dpspCas13b(AAAA)-FseI[ADAR2-DD-splitC]	196843
pzk491-pmax-NES-PUFc-FseI[ADAR2-DD-splitC]	Express NES-PUFc-FseI[ADAR2-DD-splitC]	196842
pzk488-pmax-NES-PUFc-FseI[ADAR2-DD-splitN]	Express NES-PUFc-FseI[ADAR2-DD-splitN]	196841
pmax-MCP_NES-ADARdd(E488Q)	Express MCP_NES-ADARdd(E488Q)	196840
pmax-hcoRBFOX1N-MCP-RBFOX1C	Express hcoRBFOX1N-MCP-RBFOX1C(190-397)	196839

pmax-hcoRBFOX1N-PCP-RBFOX1C	Express hcoRBFOX1N-PCP-RBFOX1C(190-397)	196838
pmax-PUFc_NES-ADARdd(E488Q)	Express PUFc_NES-ADARdd(E488Q)	196837
pzk384-pmax-PUFc-FseI-RESCUE-SalI-NES	Express PUFc-RESCUE-S	196831
pmax-hcoRBFOX1N-PUFc-RBFOX1C	Express hcoRBFOX1N-PUFc-RBFOX1C(190-397)	196830
pmax-3xFLAG_3xNLS_dPspCas13b(AAAA)_2xNLS-Clover	Express dpdpCas13b with AAAA mutation and Clover	196829
pzk408-pmax-3xflag-dpspCas13b(AAAA)-NES	Express dpdpCas13b with AAAA mutation	196828
pzk228-pmax-SgrAI[3xFLAG]-AscI-dPspCas13b(AAAA)	Express dpdpCas13b with AAAA mutation	196827
